# Chronic Variable Stress Induces Hepatic Fe(II) Deposition by Up-Regulating ZIP14 Expression via miR-181 Family Pathway in Rats

**DOI:** 10.3390/biology10070653

**Published:** 2021-07-12

**Authors:** Shuxia Jiang, Taining Guo, Shihui Guo, Jiang Gao, Yingdong Ni, Wenqiang Ma, Ruqian Zhao

**Affiliations:** 1 Key Laboratory of Animal Physiology and Biochemistry, Ministry of Agriculture and Rural Affairs, College of Veterinary Medicine, Nanjing Agricultural University, Nanjing 210095, China; 2017207003@njau.edu.cn (S.J.); 2018107012@stu.njau.edu.cn (T.G.); 2017107012@njau.edu.cn (S.G.); 2018807135@njau.edu.cn (J.G.); niyingdong@njau.edu.cn (Y.N.); zhaoruqian@njau.edu.cn (R.Z.); 2 MOE Joint International Research Laboratory of Animal Health & Food Safety, Nanjing Agricultural University, Nanjing 210095, China

**Keywords:** chronic variable stress, hepatic Fe(II), ZIP14, miR-181, rats

## Abstract

**Simple Summary:**

Modern intensive production methods attract accusations of poor animal welfare due to long-term exposure to stressors including high temperature, persistent humidity and overcrowding. Stress can be defined as any condition that threatens the physiological homoeostasis and hypothalamic-pituitary-adrenal (HPA) axis responses that tend to restore the prior stable status of the organism. Uncontrollable and unpredictable sources of stress can cause various forms of damage to the liver, which is the central mediator of systemic iron balance. Iron, notably, is an essential element for maintaining health in virtually all organisms. We found that chronic variable stress can cause weight loss and disorders of the liver iron metabolism in rats, thereby triggering liver oxidative damage. Our results also suggest that the miR-181 family is a potential target for treating iron overload-associated diseases.

**Abstract:**

It is well-known that hepatic iron dysregulation, which is harmful to health, can be caused by stress. The aim of the study was to evaluate chronic variable stress (CVS) on liver damage, hepatic ferrous iron deposition and its molecular regulatory mechanism in rats. Sprague Dawley rats at seven weeks of age were randomly divided into two groups: a control group (Con) and a CVS group. CVS reduces body weight, but increases the liver-to-body weight ratio. The exposure of rats to CVS increased plasma aspartate aminotransferase (AST), alkaline phosphatase (ALP) and hepatic malondialdehyde (MDA) levels, but decreased glutathione peroxidase (GSH-Px) activity, resulting in liver damage. CVS lowered the total amount of hepatic iron content, but induced hepatic Fe(II) accumulation. CVS up-regulated the expression of transferrin receptor 1 (TFR1) and ZRT/IRT-like protein 14 (ZIP14), but down-regulated ferritin and miR-181 family members. In addition, miR-181 family expression was found to regulate ZIP14 expression in HEK-293T cells by the dual-luciferase reporter system. These results indicate that CVS results in liver damage and induces hepatic Fe(II) accumulation, which is closely associated with the up-regulation of ZIP14 expression via the miR-181 family pathway.

## 1. Introduction

Iron is an essential nutrient for all living organisms, playing a vital role in a variety of integrative metabolic processes [1,2,3]. The iron concentration in the liver, the largest iron storage organ, is considered to be a reliable indicator of systemic iron content [4]. Dysregulation of iron homeostasis has been found in various chronic liver diseases, and this imbalance leads to iron toxicity and liver damage [5,6,7]. In particular, it has been reported that iron deficiency reduces hepatic iron and serum ferritin levels, leading to maternal hypothrombinemia in pregnant rats [5]. Mutations in transferrin receptor (TFR) 2 and hemochromatosis protein lead to severe hepatic iron deposition, lipid peroxidation, and portal fibrosis in mouse models of hereditary hemochromatosis [6]. In addition, inhibition of hepatic hepcidin expression induces significant iron deposition and subsequent liver damage in patients with chronic hepatitis C virus infections [7]. The homeostasis of hepatic iron is, therefore, tightly regulated to prevent liver damage.

Hepatic iron homeostasis is delicately controlled by diverse iron uptake and export mechanisms. The liver acquires iron primarily through transferrin-bound iron (TBI) and non-transferrin bound iron (NTBI) pathways at the hepatocyte membrane [8]. TFR1/2 delivers TBI into the hepatocytes via a classical system involving a receptor-mediated endocytosis system [9]. On the other hand, NTBI uptake on the surface of hepatocytes is mainly mediated by ZRT/IRT-like protein 14 (ZIP14, SLC39A14) [10,11,12]. Iron export is regulated by hepcidin, a liver-secreted iron-regulatory hormone that degrades ferroportin (FPN), the only known hepatocyte protein with cellular iron exporter activity [13,14]. Importantly, chronic liver disease has been proven to be closely related to the abnormal regulation of genes that are related to hepatic iron homeostasis [6,11,15,16].

Stress is a state of physiological or psychological responses induced by stimuli that threaten bodily homeostasis [17], and uncontrollable and unpredictable stressors are linked closely to various liver disorders [18]. Several studies have suggested that stress disturbs hepatic iron metabolism in rats. Heat stress, for example, significantly increases the hepatic iron content, resulting in oxidative injury in the livers of elderly rats [19]. Another recent study of rats with psychological stress revealed significantly higher activity of the interleukin-6-hepcidin axis, resulting in hepatic iron accumulation and hypoferremia [20]. He et al. discovered that the expression of hepatic iron regulatory protein 1 and TFR1 were markedly up-regulated after 7 days of injection of the stress hormone corticosterone into the tail veins of rats, eventually leading to iron accumulation in the liver [21]. Similarly, a potential link between inhibited hepatic TFR1 protein expression and reduced hepatic iron storage was identified by Li et al. [22] upon long-term exposure of rats to dexamethasone, a stress hormone analogue. Therefore, the influence of stress on hepatic iron metabolism may be closely associated with the duration, type, and intensity of the stressors [23,24].

A particularly important form of stress is the chronic unpredictable stress paradigm, which is a widely used and well-validated animal model of depression [25]. Exposure to chronic unpredictable stress has a few negative consequences for animal health, including an increased risk of metabolic disorders [26] and cardiovascular [27] and neurodegenerative diseases [28]. Importantly, the homeostatic imbalance of intracellular iron load is positively correlated with the development of these diseases. However, the possible molecular regulatory mechanism of hepatic iron deposition metabolism induced by chronic unpredictable stress have not yet been elucidated. The aim of the current study, therefore, was to investigate the effects of chronic unpredictable stress on liver damage, on the deposition of hepatic iron and on the molecular mechanisms of iron regulation in rats.

## 2. Materials and Methods

### 2.1. Animals

Sprague Dawley rats (7 week-old) weighing 225 to 250 g were purchased from Nanjing Medical University (Nanjing, Jiangsu, China). All rats were housed under standard conditions (22 ± 1 °C under a 12 h light−dark cycle) with free access to deionized water and food, except for the stressed group during the period of stress. Body weight and feed intake were recorded every 3 days for subsequent calculations of average daily gain, feed intake and iron intake. At the end of day 22, the rats were weighed and anesthetized with 20% sodium pentobarbital at a dose of 40 mg/kg. The abdominal cavity was opened, and whole blood was collected in tubes with EDTA to obtain plasma samples from the abdominal aorta. The liver was gently separated and weighed with autoclaved forceps. Animal procedures and experimental protocols were approved by the Animal Ethics Committee of Nanjing Agricultural University and the “Guidelines on Ethical Treatment of Experimental Animals” (2006) No. 398 as established by the Ministry of Science and Technology, China.

### 2.2. Induction of Chronic Variable Stress 

After a one-week adaptation, twenty-four rats were randomly distributed into two groups (*n* = 12): a control group (Con) and a chronic variable stress group (CVS). The CVS protocol was performed as previously described by Herbet et al. [29] and Jelenik et al. [30] with some modifications in the types of stressors applied. The following stressors were used: (i) restraint stress (3 h in plastic restraint tubes); (ii) cold stress (1 h at 4°C, three rats per cage without bedding); (iii) rotation stress (1 h at 150 rpm on a platform shaker); (iv) warm swim (20 min at 33 ± 2 °C); (v) cold swim (10 min at 16 ± 2 °C); and (vi) water deprivation (24 h). The stressed group animals were randomly subjected to one stressor at different times each day, and 6 different stressors appeared alternately, in order to minimize predictability and adaptability. The order of daily stress is shown in Table S1.

### 2.3. Measurement of Plasma Biochemical Parameters

Plasma iron (6063-2012, Shino-test corporation, Kanagawa, Tokyo, Japan), unsaturated iron-binding capacity (UIBC) (6062-2012, Shino-test corporation, Kanagawa, Tokyo, Japan), aspartate aminotransferase (AST) (CH0105202, Maccura, Chengdu, Sichuan), alanine aminotransferase (ALT) (CH0105201, Maccura, Chengdu, Sichuan), and alkaline phosphatase (ALP) (CH0105203, Maccura, Chengdu, Sichuan) were measured using a Hitachi 7020 automatic biochemistry analyzer (Hitachi High-Tech corporation, Kanagawa, Tokyo, Japan) with commercial kits according to manufacturers’ instructions. Total iron-binding capacity (TIBC) was calculated as the sum of the plasma iron and UIBC. Transferrin saturation (TS) was calculated as follows: TS (%)  =  (plasma iron/TIBC) × 100%. 

### 2.4. Hematoxylin and Eosin Staining

The tissue of the largest lobe of the liver was fixed overnight with 4% paraformaldehyde, embedded in paraffin and sectioned (5 μm) for hematoxylin and eosin staining. Briefly, tissue sections were baked for 4 h at 70 °C after deparaffinization and hydration. The liver section samples were soaked and stained in Harris alum hematoxylin for about 5 min, and then washed in alcohol containing 0.5% hydrochloric acid for 10 s. After washing, the samples were soaked and dyed in eosin for 30 s, then dehydrated, transparent and sealed with neutral balsam. Finally, histopathological examination was performed by observing sections under a microscope.

### 2.5. Measurement of Lipid Peroxidation and Activities of Antioxidant Enzymes

Liver samples were homogenized and then centrifuged to obtain the supernatants for detection of lipid peroxidation and activities of antioxidant enzymes. The enzyme activity of hepatic superoxide dismutase (SOD), catalase (CAT), glutathione peroxidase (GSH-Px), and hepatic malondialdehyde (MDA) levels were determined with kits (A001-3, A007-1, A005 and A003-1, Jiancheng, Nanjing, China) following the manufacturer’s instructions. The values were standardized to the total protein content.

### 2.6. Determination of Feed and Liver Iron Content

Exactly 0.5 g of feed and liver were weighed and digested as previously described [22]. Total iron concentration in feed and liver was measured using a Thermo iCE-3500 graphite atomic absorption spectrophotometer (Thermo Scientific, Wilmington, DE, USA) and was expressed as μg/g wet tissue.

### 2.7. Measurement of Hepatic Fe(II) Concentration

Hepatic Fe(II) content was measured with the iron assay kit (MAK025-1KT, Sigma, St. Louis, MO, USA). Briefly, liver samples (20 mg) were lysed in 4 to 10 volumes of lysis buffer, and then centrifuged at 16,000 *g* for 10 min at 4 °C to collect the supernatants. The samples (50 μL), assay buffer (50 μL) and iron assay buffer (5 μL) were added into wells of a 96-well plate. The plate was shaken on a horizontal shaker and then incubated for 30 min at 25 °C in darkness. Finally, 100 μL of the iron probe was added to each well, the reaction was incubated at 25 °C in the dark for 60 min and the absorbances (593 nm) were determined. A standard curve was produced according to the protocol outlined by the manufacturer. The values were standardized to the total protein content.

### 2.8. RNA Isolation and mRNA Quantification

Hepatic total RNA was extracted with TRIzol reagent (15596026, Invitrogen, Carlsbad, CA, USA) following the manufacturer’s instructions. The concentration of the extracted RNA was determined with a NanoDrop 1000 spectrophotometer (Thermo Scientific, Wilmington, DE, USA). HiScript II Q RT SuperMix (R223-01, Vazyme, Nanjing, China) was used to synthesize cDNA following the manufacturer’s instructions. Diluted cDNA (1 µL, 1:20, *vol*/*vol*) was used as a template for real-time PCR in a QuantStuioTM 6 Flex Real-Time PCR system (Applied Biosystems, Foster City, CA, USA). Peptidylprolyl isomerase A (PPIA) was evaluated and chosen as a reference gene. All primers used for this experiment are listed in Table S2 and were synthesized by Generay Biotech Co., Ltd. (Shanghai, China). 

### 2.9. Protein Extraction and Western Blotting

Hepatic total protein was extracted as described previously, with some modifications [31]. Protein content was detected by BCA assay (23225, Thermo Scientific, Waltham, PA, USA) following the manufacturer’s guidelines. After denaturation, 50 μg/lane protein was loaded on 10−15% SDS-PAGE gel and transferred onto a nitrocellulose membrane (66485, Pall Corporation, Port Washington, New York, USA). The membrane was blocked in 4% skimmed milk for 2 h at room temperature and then incubated with primary antibody at 4°C overnight, after washing three times with TBST, secondary antibody at room temperature for 2 h. Detailed information regarding these antibodies is listed in Table S3. The β-Actin was used to be the loading control.

### 2.10. Prediction of miRNA Targeting ZIP14 

The miRNAs targeting the Zip14 3′UTR were predicted and screened using two miRNA prediction bioinformatics tools: TargetScan [32] and miRDB [33]. Predicted miRNAs targeting ZIP14 are presented in Table S4. A total of 10 miRNAs were screened from the overlap of 2 different prediction tools and then quantified using real-time PCR. All mature miRNA sequences of *Rattus norvegicus* were retrieved from miRBase [34].

### 2.11. Quantification of miRNA with Real-Time PCR

Analysis of miRNA was conducted according to previous descriptions [31,35]. After treating with RNase-free DNase I (2270A, Takara, Otsu, Japan), total RNA (6 μg) was polyadenylated at 37 °C for 1 h using Poly (A) Tailing Kit (AM1350, Applied Biosystems, Waltham, MA, USA), then reverse transcribed for use in qPCR. An exogenous reference (a random DNA oligonucleotide) was added to total RNA before polyadenylation to normalize miRNA expression. All miRNA primers, exogenous reference and poly (T) adapter for miRNA are shown in Table S5.

### 2.12. Cell Culture and Luciferase Reporter Assays

HEK-293T cells were purchased from Beijing Beina Chuanglian Biotechnology Institute (China), and 10 to 20 passages of cells were used in this experiment. Cells were seeded at densities of 1 × 10^5^/well into 24-well plates and cultured in Dulbecco’s modified Eagle’s medium (DMEM) (SH30243.01, Hyclone, Logan, UT, USA) supplemented with 100 IU/mL streptomycin, 100 IU/mL penicillin, and 10% fetal bovine serum (FBS) (A31608-02, Gibco, Carlsbad, CA, USA). When cells reached a confluence of 85 to 90%, lipofectamine 2000 (11668019, Life Technologies Inc., Waltham, MA, USA) was used to cotransfect the cells with the miR-181 mimics (100 nM), pmirGLO-ZIP14 3′UTR-wt (50 ng) or pmirGLO-ZIP14 3′UTR-mut (50 ng). At 48 h post-transfection, luciferase activity was detected with a GlOMAXTM 96 microplate luminometer (E1910, Promega, Madison, WI, USA). Syntheses of mimics of miR-181 family members (miR-181a-5p, miR-181b-5p, miR-181c-5p, and miR-181d-5p) were performed by Biomics Biotechnologies Co., Ltd. (Nantong, Jiangsu, China). These sequences are shown in Table S6.

### 2.13. Statistical Analysis

Data are presented as mean ± SEM. One-way ANOVA was applied in SPSS 20.0 (Chicago, IL, USA) to analyze the differences between the two groups. GraphPad Prism 5.0 was used for graphical presentations. Differences were considered statistically significant at *p* < 0.05.

## 3. Results

### 3.1. Body Weight, Liver Weight and Liver Indexes of Rats Exposed to Chronic Variable Stress

**Figure 1:**
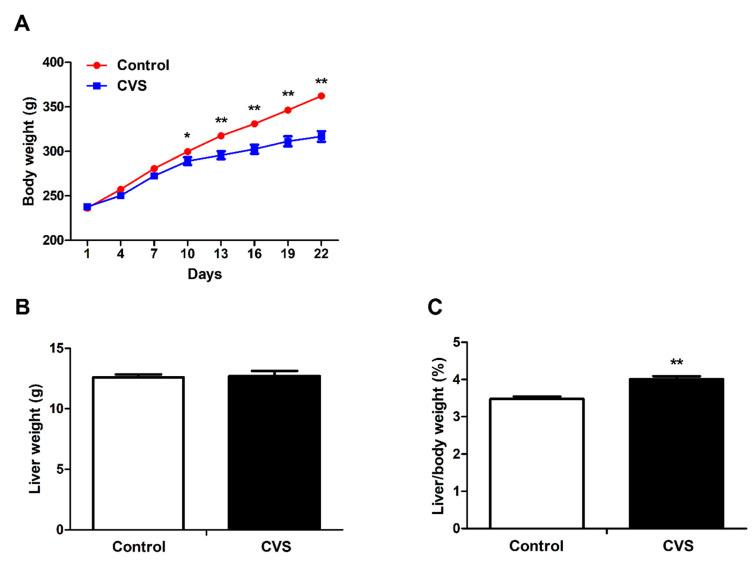
Effect of chronic variable stress on body weight and liver indexes in rats. (**A**) Body weight was calculated every three days. (**B**) Liver weight. (**C**) Liver/body weight. Con: control group; CVS: chronic variable stress group. Values are expressed as means ± SEM; *n* = 12 in each group. * *p* < 0.05, ** *p* < 0.01.

Chronic variable stress was associated with a significantly reduced body weight (*p* < 0.01, Figure 1A) of the rats. The liver weight remained unchanged between the groups (Figure 1B); therefore, stress was associated with a significantly increased ratio of liver weight to body weight (*p* < 0.01, Figure 1C). Moreover, the average daily feed intake (*p* < 0.05), average daily gain (*p* < 0.01), and average daily iron intake (*p* < 0.05) were significantly suppressed in chronic variable stress-loaded rats (Table S7).

### 3.2. Indicators and Histomorphology Related to Liver Injury of Rats Exposed to Chronic Variable Stress

**Figure 2:**
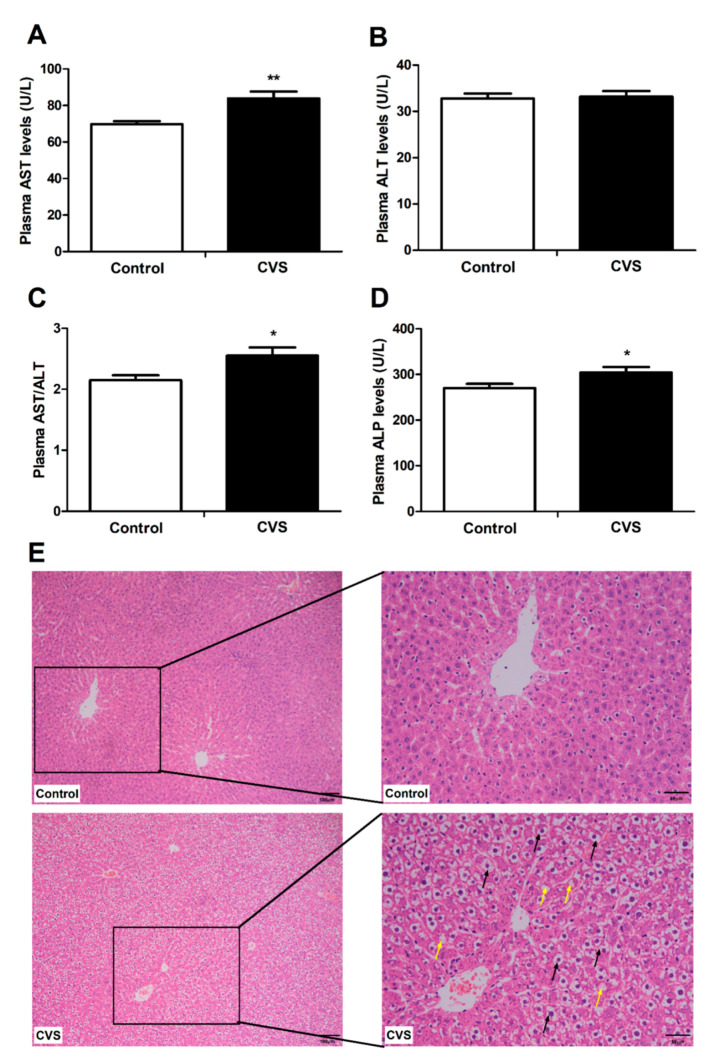
Effect of chronic variable stress on plasma indicators and histomorphology related to liver injury in rats. (**A**–**D**) Plasma AST, ALT, AST/ALT, and ALP enzyme levels were determined as described in Materials and Methods. (**E**) Hematoxylin and eosin-stained liver tissue was observed at magnifications of ×100 and ×200. Black arrows indicate hepatocyte swelling; yellow arrows indicate hepatocyte vacuolation. Con: control group; CVS: chronic variable stress roup. Values were expressed as means ± SEM; *n* = 12 in each group for panels **A**–**D**; *n* = 3 in each group for panel **E**. * *p* < 0.05, ** *p* < 0.01.

Chronic variable stress significantly increased the plasma AST (*p* < 0.01, Figure 2A), AST/ALT (*p* < 0.05, Figure 2C), and ALP (*p* < 0.05, Figure 2D) levels, but it did not affect the plasma ALT level (Figure 2B). In addition, the hepatocytes from the chronic variable stress-loaded rats were swollen and vacuolated, and the cytoplasm was sparse, relative to the hepatocytes from the control animals (Figure 2E).

### 3.3. Hepatic Lipid Peroxidation and Activity of Antioxidant Enzyme Activities of Rats Exposed to Chronic Variable Stress

**Figure 3:**
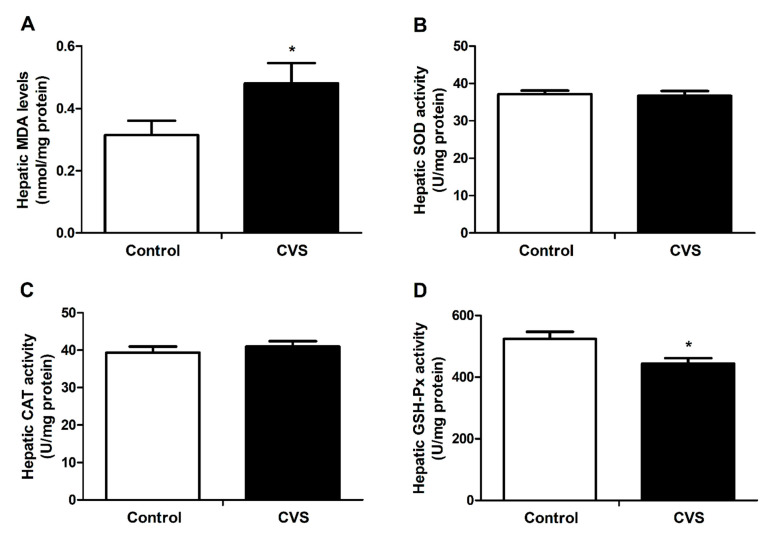
Effect of chronic variable stress on hepatic lipid peroxidation and antioxidant enzyme activities in rats. (**A**) The MDA level and (**B**–**D**) SOD, CAT and GSH-Px activities were determined as described in Materials and Methods. Con: control group; CVS: chronic variable stress group. Values were expressed as means ± SEM; *n* = 12 in each group. * *p* < 0.05.

As shown in Figure 3A, the hepatic MDA level was significantly increased in chronic variable stress-loaded rats (*p* < 0.05), and the hepatic GSH-Px activity was markedly declined in the rats that had suffered with chronic variable stress (*p* < 0.05, Figure 3D). The SOD and CAT activities between the two groups were not obviously different (Figure 3B,C).

### 3.4. Levels of Hepatic Iron and Iron-Related Genes and Proteins in Rats Exposed to Chronic Variable Stress

**Figure 4:**
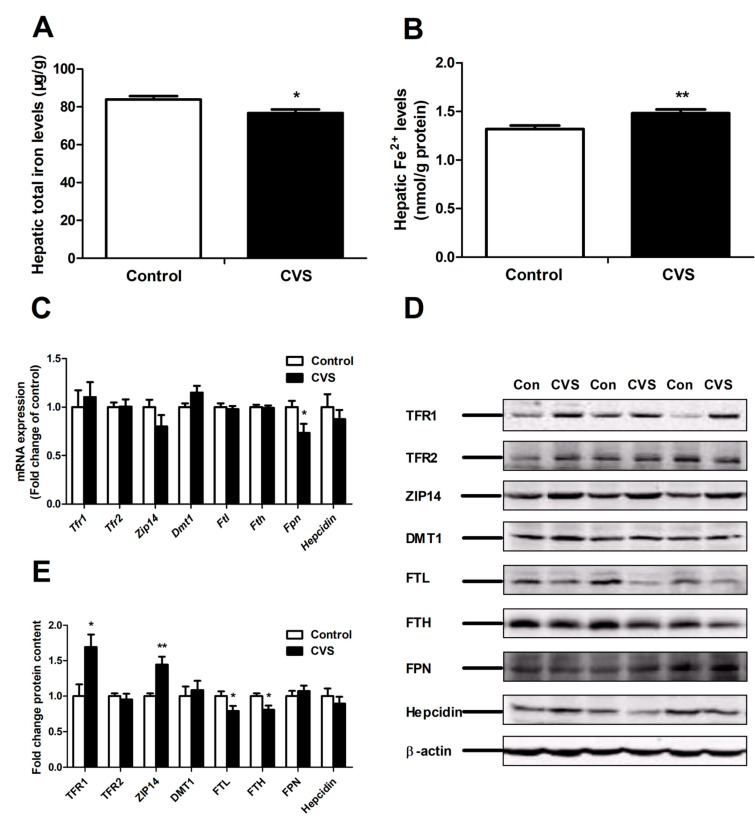
Effect of chronic variable stress on hepatic iron level and expression of iron metabolism-related genes and proteins. (**A**) Hepatic iron deposition. (**B**) Hepatic Fe^2+^ level. (**C**) Expression of *Tfr1*, *Tfr2*, *Zip14*, *Dmt1*, *Ftl*, *Fth, Fpn* and *Hepcidin* mRNA were evaluated using qRT-PCR. (**D**) Representative Western blot analyses of TFR1, TFR2, ZIP14, DMT1, FTL, FTH, FPN and Hepcidin protein expression. (**E**) Densitometric analysis of Western blot results showing TFR1, TFR2, ZIP14, DMT1, FTH, FTL, FPN and Hepcidin protein levels. Con: control group; CVS: chronic variable stress group. Values were expressed as means ± SEM; *n* = 12 in each group for panels A,B; *n* = 7 in each group for panel C; *n* = 7 in each group for panel D. * *p* < 0.05, ** *p* < 0.01.

Chronic variable stress reduced hepatic iron deposition (*p* < 0.05, Figure 4A), but increased hepatic Fe(II) levels (*p* < 0.01, Figure 4B). The expression of several hepatic iron metabolism-related genes (*Tfr1*, *Tfr2*, *Zip14*, *Dmt1*, *Ftl*, *Fth* and *Hepcidin*) remained unchanged; however, the expression of *Fpn* was significantly decreased (*p* < 0.05) in chronic variable stress-loaded rats (Figure 4C). Chronic variable stress correlated with an up-regulation of the protein expression of TFR1 (*p* < 0.05) and ZIP14 (*p* < 0.01), but with a down-regulation of the expression of iron storage-related proteins FTL (*p* < 0.05) and FTH (*p* < 0.05) (Figure 4D,E). In addition, chronic variable stress resulted in an increase in plasma iron levels, total iron binding capacity and transferrin saturation (Table S8).

### 3.5. Hepatic Expression and Functional Validation of miRNAs Targeting Zip14

**Figure 5:**
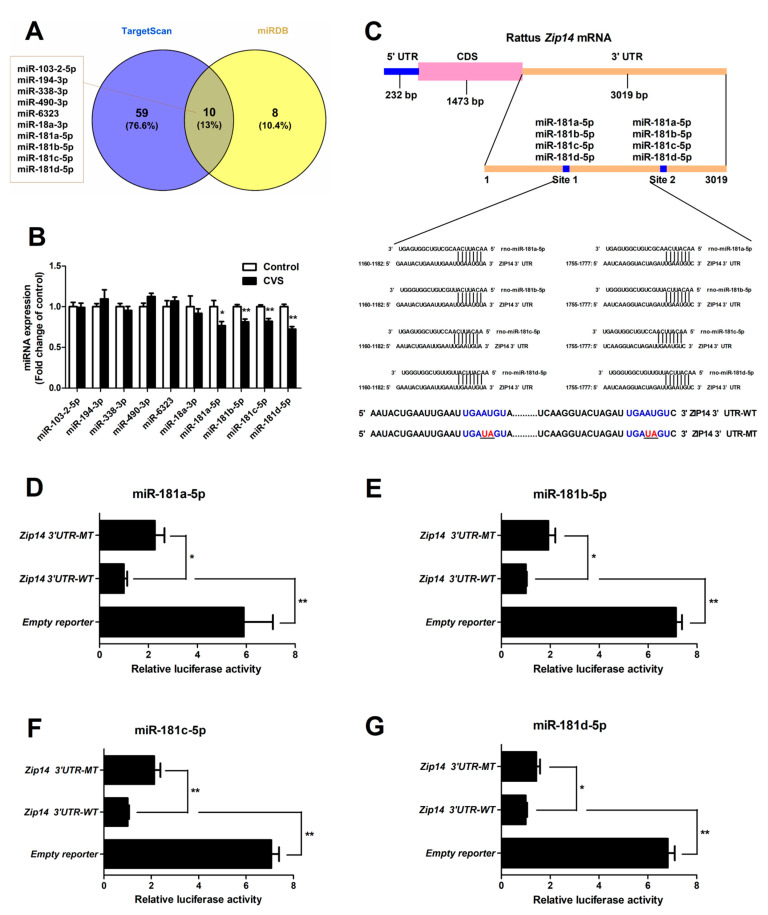
miR-181 family members directly target the 3′UTR of *Zip14*. (**A**) Venn diagrams showing 10 overlapping miRNAs identified by TargetScan and miRDB. (**B**) Expression levels of candidate *Zip14* gene 3′UTR-targeting miRNAs were analyzed by qRT-PCR. (**C**) Schematic description of conserved binding sites for miR-181 in *Zip14* mRNA and sequence of the wild-type and mutated miR-181 binding site. (**D**–**G**) Relative luciferase activities of wild-type and mutated *Zip14* 3′UTR reporter plasmid in HEK-293T cells cotransfected with an miR-181 overexpressing plasmid for 24 h. Values were expressed as means ± SEM, *n* = 6 in each group for panel B, *n* = 4 in each group for panels D-G. * *p* < 0.05, ** *p* < 0.01.

Ten miRNAs targeting *Zip14* were identified by TargetScan and miRDB (Figure 5A). The expression of hepatic miR-181 family members (miR-181a-5p, miR-181b-5p, miR-181c-5p and miR-181d-5p) were significantly decreased in chronic variable stress-treated rats (*p* < 0.05 or *p* < 0.01, Figure 5B). Bioinformatics analyses revealed the seed sequence of miR-181 family members and its target sites located in the *Zip14* 3’UTR (Figure 5C). A dual-luciferase reporter assay revealed that mutations of miR-181 binding sites resulted in a significant enhancement (*p* < 0.01 or *p* < 0.05) of the luciferase reporter activity (Figure 5D–G), which supported the direct regulatory role of miR-181 on *Zip14*.

## 4. Discussion

In this study, we found that chronic variable stress could inhibit the feed intake and growth of rats. This finding is consistent with accumulating evidence that suggests that both long-term dexamethasone and mild chronic intermittent cold exposure result in a reduction of feed intake and body weight in rats [22,36]. In addition, the importance of chronic stress has been suggested by experiments in which a single injection of the stress hormone dexamethasone did not affect feed intake in rats, but where feed intake and weight gain were inhibited dose-dependently by continuous daily injections of dexamethasone [37]. Interestingly, it has been noted that unpredictable or acute stress leads to a great reduction in feed intake and body weight, whereas chronic social stress results in an increase in caloric intake and weight gain [38,39,40]. The decline of feed intake induced by unpredictable stress is closely linked to the unpredictability and uncontrollability of stimuli; on the other hand, the adaptive response or habituation is induced by predictable stress [41].

High levels of AST and ALP are considered to be important markers of hepatotoxicity, as they are released into the extracellular fluid by the damaged liver [42,43]. High levels of AST and/or ALP have been reported to be induced by multiple stress responses, suggesting that the liver is a primary target of stress-induced damage. For example, exposure of rats to acute cold-restraint stress significantly enhances serum AST levels [44]. In addition, both restraint stress and CCl_4_ administration induce significant hepatic damage in rats, as identified by increased activities of serum AST and ALP [42,45]. Stress induction via immobilization also leads to the development of a significant enhancement in the activity of plasma AST [46]. Consistent with previous results, then, we found that chronic variable stress also resulted in hepatic damage, which was confirmed by increased plasma AST and ALP activity and histological observation in rats. 

Hepatic damage induced by stress is related to disorders of antioxidant systems [46,47]. To investigate the impact of chronic stress on the liver, it is therefore important to identify the markers of oxidative stress. MDA is the end product of lipid peroxidation, which is an indicator of tissue damage caused by oxidative stress [48]. GSH-Px is an intracellular antioxidant enzyme that degrades lipid hydroperoxides into their corresponding alcohols. Therefore, estimating the activity of GSH-Px is an appropriate indirect approach to evaluate the pro-oxidant−antioxidant effect in tissues [49]. Accordingly, our data that demonstrated both increased MDA and decreased GSH-Px activity in the liver indicated that chronic variable stress likely leads to hepatic oxidative stress. 

Recent studies indicate that iron toxicity and oxidative stress depend on the amount of “free” iron rather than the total iron load [50,51]. It has been shown, for instance, that free iron is associated with levels of MDA, a biomarker of oxidative stress [52]. Similarly, bioimaging and biosensing have also revealed that increasing concentrations of hepatic Fe(II) were linked closely to oxidative stress [53]. Free ferrous iron (Fe(II)) is toxic, and it is recognized as a mediator of tissue damage through accelerating the Fenton reaction to catalyze the formation of reactive radicals [54,55]. 

Homeostasis of intracellular Fe(II) is regulated by many factors involved in iron uptake, storage and excretion. NTBI (Fe(II)) is rapidly taken up by the liver via the ZIP14 pathway, whereas TBI is incorporated through TfR-mediated endocytosis. In endosomes, reduced Fe(II) is released into the cytosol via the DMT1 pathway [56]. Under normal circumstances, excess Fe(II) is stored in ferritin after oxidization, or exported to the circulation by its exporter FPN. Interestingly, an important finding of the present study was that chronic variable stress increased hepatic Fe(II) concentrations, but it decreased the total amount of hepatic iron. Here, chronic variable stress induced Fe(II) deposition, increased ZIP14 expression and decreased the expression of ferritin (as FTL and FTH), but it did not affect the expression of DMT1 or FPN at the protein level. Therefore, the up-regulation of ZIP14 suggests that uptake of Fe(II) plays the key role in hepatic Fe(II) accumulation upon exposure to chronic variable stress. 

ZIP14, a member of the SLC39A family of metal-ion transporters that is abundantly expressed in the liver, transports Fe(II) from the blood into hepatocytes [10,12]. ZIP14 deficiency markedly reduces Fe(II) deposition in the hepatocytes of hemochromatosis mice [11]. ZIP14 overexpression leads to an increase of Fe(II) uptake in human beta-cells [57]. In addition, hemochromatosis also inhibits Fe(II) uptake via reducing the expression of ZIP14 in HepG2 cells [58]. Our results showed that chronic variable stress dramatically elevated ZIP14 expression at the protein level, but it did not affect the mRNA expression of *Zip14*. The inconsistency between mRNA and protein expression suggests the possibility of post-transcriptional regulation involving ZIP14.

One candidate for molecules that might be involved in such post-transcriptional regulation are miRNAs. As small non-coding RNAs, miRNAs play a pivotal role in post-transcriptional regulation and drive transcriptional inhibition or mRNA degradation through binding to the 3′UTR on a target mRNA [59,60]. Numerous miRNAs have been reported to regulate iron uptake and export proteins and have been shown to play vital roles in maintaining iron homeostasis [61]. Members of the miRNA-16 family and miR-let-7d inhibit iron uptake protein-DMT1 expression by targeting its 3′UTR in K562 and HCT116 cells, respectively [31,62]. Decreasing miR-148a expression induced high expression of TFR1, resulting in increased cellular iron and cell proliferation in hepatocellular carcinoma [63]. Two other miRNAs, miR-20a and miR-20b, suppress FPN expression in lung cancer and intestinal cells, regulating intracellular iron export [35,64]. The present results indicated that the expression of the hepatic miR-181 family members was significantly impaired in chronic variable stress-loaded rats. The dual-luciferase reporter assay is a useful tool to verify miRNA−mRNA interactions, and it provided strong evidence that miRNAs play a key role in the control of the expression of a target gene through binding to its 3′UTR [65,66]. The dual-luciferase reporter assays further revealed that these miR-181 family members directly target the 3′UTR of *Zip14* mRNA.

## 5. Conclusions

Taken together, these results demonstrate that chronic variable stress induces liver damage and elevated hepatic Fe(II) content, which is closely associated with the up-regulation of ZIP14 expression via the miR-181 family pathway. These finding provides a further understanding of hepatic disorders of iron metabolism induced by chronic variable stress.

## Data Availability

Not applicable.

## Supplementary Materials

The following are available online at https://www.mdpi.com/article/10.3390/biology10070653/s1. Table S1, Stressor agents used during the chronic variable stress. Table S2, Nucleotide sequences of specific primers for target genes; Table S3, Details of antibodies used in the experiments; Table S4, Prediction of miRNAs targeting ZIP14 using TargetScan and miRDB; Table S5, Sequences of miRNA and the corresponding primers; Table S6, Sequences of miRNA mimics; Table S7, The effect of chronic variable stress on average daily gain, average daily feed intake and average iron intake of rats; Table S8, Effect of chronic variable stress on plasma iron metabolism-related parameters in rats.
