# Supplementary material for: Chronic Variable Stress Induces Hepatic Fe(II) Deposition by Up-Regulating ZIP14 Expression via miR-181 Family Pathway in Rats
Source: Biology (Basel). 2021 Jul 12;10(7):653. doi: 10.3390/biology10070653 (PMC8301360; doi:10.3390/biology10070653)
Supplement: Supplementary file 1 [file biology-10-00653-s001.zip › biology-1247928-supplementary.pdf]

Supplementary materials

Supplementary materials contain Supplementary Tables 1-8.

Table S1. Stressor agents used during the chronic variable stress.

Day of treatment	Stressor	Duration	Start time
1	restrain stress	3 h	9:00 am
2	cold stress	1 h	10:00 am
3	rotation stress	1 h	9:00 am
4	warm swim	20 min	10:30 am
5	cold swim	10 min	11:am
6	Water depriva-tion	24 h	10:00 am
7	rotation stress	1 h	9:00 am
8	restrain stress	3 h	9:00 am
9	cold swim	10 min	11:am
10	warm swim	20 min	10:30 am
11	Water depriva-tion	24 h	10:00 am
12	cold stress	1 h	10:00 am
13	cold swim	10 min	11:am
14	warm swim	20 min	10:30 am
15	rotation stress	1 h	9:00 am
16	Water depriva-tion	24 h	10:00 am
17	cold stress	1 h	10:00 am
18	restrain stress	3 h	9:00 am
19	rotation stress	1 h	9:00 am
20	warm swim	20 min	10:30 am
21	restrain stress	3 h	9:00 am

Table S2. Nucleotide sequences of specific primers for target genes.

**Target genes**	**Sequences (5’ to 3’)**		**Products**	**Accession No.**
Tfr1	F: tttagtgattgttagagcagggaa	R: atggcggaaactgagtatgg	212 bp	NM_022712.1
Tfr2	F: tccctccagtagaatcatcgg	R: tgttgaccacaaggcgtaa	180 bp	NM_001105916.1
Dmt1	F: ttgtcgtctccgtctttgct	R: ccagggtagagttgtcgttagg	120 bp	NM_013173.2
Zip14	F: atcccagagtgcttgctac	R: ctaatgccttggaagacaga	153 bp	NM_001107275.1
Ftl	F: tccaggatgtgcagaagcc	R: ccctacggaggttggtcag	228 bp	NM_022500.4
Fth	F: ggaacttcacaaactggctac	R: tggatttcacctgctcatt	89 bp	NM_012848.2
Fpn	F: agtcattggctgtggtttc	R: tcaagttcacggatgttaga	228 bp	NM_133315.2
Hepcidin	F: ctgcctgtctcctgcttctc	R: agttggtgtctcgcttcctt	162 bp	NM_053469.1
Ppia	F: ttgggaaggtgaaagaaggc	R: gttgtccacagtcggagatg	105 bp	NM_017101.1

Note: Tfr1, transferrin receptor 1; Tfr2, transferrin receptor 2; Dmt1, divalent metal transporter 1; Zip14, ZRT/IRT-like protein 14; Ftl, ferritin light chain; Fth, ferritin heavy chain; Fpn, ferroportin; Ppia, peptidylprolyl isomerase A.

Table S3. Details of antibodies used in the experiment.

Antibodies	MW	Species	Source	Catalogue no.	Dilution ratio
Primary antibody					
TFR1	90 kd	Mouse	Life Technologies	136800	1:1000
TFR2	98 kd	Rabbit	Sigma	SAB2700078	1:1000
DMT1	72 kd	Rabbit	Proteintech	20507-1-AP	1:1000
ZIP14	54 kd	Rabbit	Abcam	ab123988	1:500
FTL	19 kd	Rabbit	Bioworld	BS7403	1:1000
FTH	21 kd	Rabbit	Affinity Biosciences	DF6278	1:1000
FPN	53 kd	Goat	Santa Cruz	sc-49668	1:500
Hepcidin	32 kd	Rabbit	Abcam	ab75883	1:500
β-actin	43 kd	Mouse	Bioworld	BS6007M	1:10000
Second antibody					
Goat anti-Mouse IgG (H+L) -HRP		Goat	Bioworld	BS12478	1:10000
Goat anti-Rabbit IgG (H+L)-HRP		Goat	Bioworld	BS13278	1:10000
Rabbit Anti-Goat IgG (H+L)-HRP		Rabbit	Bioworld	BS30503	1:10000

Note: TFR1, transferrin receptor 1; TFR2, transferrin receptor 2; DMT1, divalent metal transporter 1; ZIP14, ZRT/IRT-like protein 14; FTL, ferritin light chain; FTH, ferritin heavy chain; FPN, ferroportin.

Table S4. miRNAs targeted ZIP14 prediction using TargetScan and miRDB.

NO.	TargetScan	miRDB
1	**miR-18a-3p**	miR-466d
2	miR-1912-3p	**miR-338-3p**
3	**miR-194-3p**	miR-153-5p
4	miR-667-3p	**miR-6323**
5	miR-218a-5p	miR-195-3p
6	miR-3594-5p	miR-653-5p
7	miR-448-5p	**miR-18a-3p**
8	miR-3084c-3p	miR-298-3p
9	miR-192-3p	**miR-103-2-5p**
10	miR-3075	**miR-194-3p**
11	miR-1188-3p	miR-149-5p
12	miR-344a-5p	**miR-181c-5p**
13	miR-301a-5p	**miR-181a-5p**
14	miR-3590-5p	**miR-181d-5p**
15	**miR-338-3p**	miR-466b-5p
16	miR-301b-5p	**miR-181b-5p**
17	**miR-103-2-5p**	miR-466c-5p
18	miR-711	**miR-490-3p**
19	miR-6334	
20	miR-758-3p	
21	miR-425-5p	
22	miR-107-5p	
23	miR-484	
24	miR-99a-3p	
25	miR-200b-5p	
26	miR-210-5p	
27	miR-489-3p	
28	miR-541-3p	
29	miR-16-5p	
30	miR-195-5p	

31	miR-10b-3p
32	miR-292-3p
33	**miR-6323**
34	miR-200a-5p
35	miR-103-1-5p
36	miR-3571
37	let-7c-1-3p
38	miR-3561-5p
39	miR-99b-3p
40	miR-878
41	miR-196c-3p
42	miR-485-5p
43	miR-217-3p
44	miR-3561-3p
45	miR-15a-5p
46	miR-204-3p
47	miR-15b-5p
48	miR-497-5p
49	miR-3085
50	miR-208b-5p
51	miR-1199-5p
52	miR-540-5p
53	miR-3586-5p
54	miR-339-5p
55	miR-322-5p
56	miR-3570
57	**miR-181b-5p**
58	**miR-181d-5p**
59	**miR-181a-5p**
60	**miR-181c-5p**
61	miR-3560
62	**miR-490-3p**

63	miR-135b-5p
64	miR-1188-5p
65	miR-881-3p
66	miR-770-5p
67	miR-134-3p
68	miR-296-3p
69	miR-208a-5p

Note: The microRNAs predicted to target *Zip14* are shown using four prediction tools including TargetScan (Context++ Score ≥ 85) and miRDB(Target Score≥ 60). In bold are overlapping microRNAs of the prediction results.

Table S5. miRNA and the corresponding primer sequences.

**Names**	**Sequence (5'to 3')**	**Accession No.**
miR-103-2-5p	agcttctttacagtgct	MIMAT0017113
miR-194-3p	ccagtggggctgctgttatct	MIMAT0017148
miR-338-3p	tccagcatcagtgattttgttga	MIMAT0000581
miR-490-3p	caacctggaggactccatgctg	MIMAT0012823
miR-6323	tggaactccatctctggtctgtgca	MIMAT0025062
miR-18a-3p	actgccctaagtgctccttct	MIMAT0017095
miR-181a-5p	aacattcaacgctgtcggtgagt	MIMAT0000858
miR-181b-5p	aacattcattgctgtcggtgggt	MIMAT0000859
miR-181c-5p	aacattcaacctgtcggtgagt	MIMAT0000857
miR-181d-5p	aacattcattgttgtcggtgggt	MIMAT0005299
Universal primer	tagagtgagtgtagcgagca	N/A
Poly(T) adapter	tagagtgagtgtagcgagcacagaattaatacgactcactatag g(t)16vn	N/A
Exogenous reference	gtgacccacgatgtgtattcgc	N/A

Table S6. miRNA mimics sequences.

Names	Sequence (5'to 3')	Source	Catalogue no.
miR-181a-5p mimics	aacauucaacgcugucggugagu	Biomics Biotech	BG091801
miR-181b-5p mimics	aacauucauuguugucggugggu	Biomics Biotech	BG0708
miR-181c-5p mimics	aacauucaaccugucggugagu	Biomics Biotech	BG091801
miR-181d-5p mimics	aacauucauuguugucggugggu	Biomics Biotech	BG0708

Table S7. The effect of chronic variable stress on average daily gain, average daily feed intake and average iron intake of rats.

**Parameters**	**Control**	**CVS**	***P*-Value**
Average daily gain (g/d)	5.99 ± 0.09	3.76 ± 0.23	0.00
Average daily feed intake (g/d)	24.74 ± 0.37	23.50 ± 0.38	0.04
Average daily Fe intake (mg/d)	7.49 ± 0.11	7.12 ± 0.11	0.04

Note: CVS, Chronic variable stress group. Values were expressed as means ± SEM, n = 12 in each group.

Table S8. Effect of chronic variable stress on plasma iron metabolism-related parameters in rats.

**Parameters**	**Control**	**CVS**	***P*-Value**
Plasma iron (μmol/L)	23.02 ± 1.39	32.47 ± 1.43	0.00
TIBC (μmol/L)	72.36 ± 1.43	79.61 ± 1.60	0.00
UIBC (μmol/L)	49.34 ± 1.78	47.14 ± 1.60	0.37
Ts (%)	31.86 ± 1.91	40.78 ± 1.59	0.00

Note: CVS, Chronic variable stress group; TIBC, total iron binding capacity; UIBC, unsaturated iron binding capacity; TS, transferrin saturation. Values were expressed as means ± SEM, n = 12 in each group.
